# Estimating Ancestral Ranges: Testing Methods with a Clade of Neotropical Lizards (Iguania: Liolaemidae)

**DOI:** 10.1371/journal.pone.0026412

**Published:** 2011-10-20

**Authors:** Juan Manuel Díaz Gómez

**Affiliations:** Cátedra de Diversidad Biológica IV, Facultad de Ciencias Naturales, CONICET-IBIGEO, Universidad Nacional de Salta, Salta, Argentina; Smithsonian's National Zoological Park, United States of America

## Abstract

Establishing the ancestral ranges of distribution of a monophyletic clade, called the ancestral area, is one of the central objectives of historical biogeography. In this study, I used three common methodologies to establish the ancestral area of an important clade of Neotropical lizards, the family Liolaemidae. The methods used were: Fitch optimization, Weighted Ancestral Area Analysis and Dispersal-Vicariance Analysis (DIVA). A main difference from previous studies is that the areas used in the analysis are defined based on actual distributions of the species of Liolaemidae, instead of areas defined arbitrarilyor based on other taxa. The ancestral area of Liolaemidae found by Fitch optimization is Prepuna on Argentina, Central Chile and Coastal Peru. Weighted Ancestral Area Analysis found Central Chile, Coquimbo, Payunia, Austral Patagonia and Coastal Peru. Dispersal-Vicariance analysis found an ancestral area that includes almost all the areas occupied by Liolaemidae, except Atacama, Coquimbo and Austral Patagonia. The results can be resumed on two opposing hypothesis: a restricted ancestral area for the ancestor of Liolaemidae in Central Chile and Patagonia, or a widespread ancestor distributed along the Andes. Some limitations of the methods were identified, for example the excessive importance of plesiomorphic areas in the cladograms.

## Introduction

Inferring the ancestral area of distribution for a clade of organisms is one of the classic goals of historical biogeography [1], and is part of the natural history of the organisms. In studies that try to assess the relative importance of vicariance and dispersal in the distribution of a group of organisms and its speciation, an important subject is the reconstruction of the ancestral ranges of distribution for the taxa analyzed [2].

Historical biogeography deals with two kinds of problems, as pointed by Hovenkamp [3]: Earth history and Taxon history. The first approach attempts to establish area relationships based on the phylogenies of at least two taxa inhabiting the areas of interest. The taxon-history approach seeks to elucidate the biogeographic history of particular taxa. The utility of the latter approach has been criticized [4], [5] because inferences are restricted to general patterns. However, as noted by Bremer [6], the search for the historical biogeography of individual groups is a valid procedure, and is part of the study of the natural history of the organisms. In many cases, the main assumption of vicariance biogeography, namely that the ancestral area of a taxon is identical to the present distribution, may not apply. For example, widespread (cosmopolitan) groups consisting of many taxa of very limited distributions. If extant taxa are limited in their distribution, it does not seem probable that its common ancestor was cosmopolitan [6]. Another example is when all relatives of widespread taxa have limited distributions, as is the case of the humans and the great apes [7]. In these cases, it may seem logical to search for an ancestral area different than the sum of the individual areas of the species. As such, ancestral area analysis is not *ad hoc* or unscientific [3], but another way to make hypotheses to explain the distribution of taxa.

The main procedure to study the biogeography of individual groups is the ancestral area methodology. Ancestral area analysis was proposed by Bremer [6] as a way to identify the area of distribution of the ancestor of a monophyletic group, which he termed ancestral area.

The main assumption of the ancestral area approach is that the ancestral area of a taxon can be inferred from the topological information in its area cladogram [8], given the assumptions that (1) plesiomorphic areas in a cladogram are more likely part of the ancestral area than apomorphic areas; and (2) areas represented on more than one branch have a higher probability of being part of the ancestral area than areas less represented. For ancestral area analysis, I applied three methods: Fitch optimization [7], weighted ancestral area Analysis [8], and Dispersal-Vicariance analysis (DIVA) [9]. These methods use optimizations with reversible parsimony for estimating ancestral areas. Fitch optimization was proposed by Ronquist [7] to avoid the problems of Camin-Sokal (irreversible) parsimony originally proposed by Bremer [6]. Weighted ancestral area analysis uses Fitch parsimony with a weighting scheme that weights favorably plesiomorphic, and more common areas. With this method, a probability index (PI) is calculated to give a measure of the likelihood of a particular area being part of the ancestral area. DIVA searches ancestral areas using a three-dimensional cost matrix that gives different costs to events, minimizing the dispersal events needed for explaining the distributions. Using this approach, vicariance events have no cost, whereas dispersals and extinctions have a cost of one per area unit added to the distribution. The optimal reconstruction(s) are those requiring the minimal number of dispersal events.

Ancestral area methods have been criticized, mainly on the basis that these methods are strictly a dispersalist approach [10], [11], or because of their basic assumption, namely that more plesiomorphic areas will be more likely to be part of the ancestral area, comparing it to the progression rule of Hennig [12], or because of the impossibility of identifying one basal area in a symmetrical cladogram [13], [14], [15]. Dispersal-Vicariance Analysis has also been criticized for its bias towards an all-vicariance explanation [16], and for its inability to model extinction and range expansions [17].

Recently, a new method for estimating geographic range evolution [18], [19] named Dispersal-extinction-cladogenesis model (DEC) have been proposed. This method enables the inference of ancestral ranges in a likelihood framework, range contractions and expansions are caused by dispersal to an unoccupied area and local extinction within an area. This method requires an explicit description of likelihood of dispersal between areas and estimates of lineage divergence times. Given a phylogeny, the distribution of the taxa involved, and an explicit model of Dispersal-extinctin and cladogenesis, dispersal and extinction rates are calculed using maximum likelihood.

Ancestral area methods however, despite all its shortcomings, remain being widely used as ways to infer the history of a taxon. DIVA in particular, remain very popular in the literature (more than 340 citations since it was published [17]), hence, it is very important to evaluate the behavior of the more common methods used for reconstructing ancestral ranges.

### Liolaemidae

Liolaemid lizards are the most common reptiles of southern South America. Members of this clade are distributed from the high Andes of central Peru to the shores of Tierra del Fuego and from sea level to more than 5000 m [19]–[24]. Liolaemidae consists of three genera: *Ctenoblepharys*, *Liolaemus*, and *Phymaturus* [25]–[28], which currently include approximately 240 species [23], [28], [29]. The monotypic *Ctenoblepharys* is known only from coastal southern Peru [26 and is the sister taxon of the clade *Liolaemus* plus *Phymaturus* [28], [29]. *Phymaturus* are robust, saxicolous lizards, which are distributed from the high Andes of western Argentina and eastern Chile, to the Patagonian tablelands of Argentina [24]. *Liolaemus* is the most diverse genus of lizards in the southern hemisphere, include 223 recognized species (second only to *Anolis* in species richness), and an average of 4–5 new species are described per year [23], [30], [31].

Despite the importance of the group and the publication of several recent phylogenies, there are few explicit, quantitative studies dealing with its historical biogeography. Cei [32] characterized the Patagonia as an active centre for speciation and dispersal for the Patagonian herpetofauna, including *Liolaemus* as an example of recent adaptive radiation. Later [21], an Andean-Patagonian origin was proposed for *Phymaturus*, based on the refuge theory for geographic speciation, where the patagonic tablelands would have acted as refuges and neo-dispersal centres [33]. Pereyra [34] based on a phenetic analysis using meristic and chromosomic data, supported a dispersal scenario proposed by Cei [35] that placed Patagonia as the centre of origin for *Phymaturus* with the northern range of *Phymaturus* in Catamarca province, Argentina, where it is ecologically replaced by a species of *Liolaemus* which inhabits similar rocky habitats as *Phymaturus*. This scenario assumes that the southern populations of *Phymaturus* would have experienced more drastic climatic and vegetation changes than the northern populations, which would have caused extinctions of several of the original southern populations. Recently, Lobo & Quinteros [24] studied the historical biogeography of *Phymaturus*, assigning to their terminal taxa the areas proposed by other authors [36]–[38]. They discussed the congruence of the various phylogenies that they obtained with the area cladograms of the aforementioned authors. They also compared the area relationships inferred from the *Phymaturus* phylogenies with the biogeographic analysis of the relationships between provinces of the Andean subregion made by Morrone [39], [40]. However, they did not perform any formal biogeographic analysis. Díaz Gómez [16] made the first analysis of the historical biogeography of *Phymaturus* using quantitative methodology. In that study, an ancestral area analysis was made on a tree that also included *Ctenoblepharys* and a single terminal representing *Liolaemus*, using three different methods of analysis. However, the areas used in that study were previously defined, and based on the distribution of arthropods [36]–[38].

In the case of *Liolaemus*, there have been biogeographic studies using formal analyses: Young-Downey [41] made a Brooks Parsimony Analysis (BPA) on a phylogeny of *Liolaemus*; Lobo [42] in a phylogenetic analysis of the Chilean group of *Liolaemus* assigned the areas defined by Roig-Juñent [43] to the species listed as terminals; Schulte *et al.* [44] optimized the distribution of species of *Liolaemus* on a molecular phylogeny, making an ancestral area analysis, although it was not explicit. Díaz Gómez & Lobo [45] made an ancestral area analysis of the Chilean group of *Liolaemus* using Fitch optimization, Weighted Ancestral Area Analysis and Dispersal-Vicariance analysis (DIVA).

A common problem of all those studies is that the areas used as units for the analyses were based on other taxa's distribution [16], [24], [42], [45], or arbitrarily defined [44]. As a result, the areas may not describe adequately the distribution of Liolaemid lizards. Most of these lizards have restricted distributions, or are endemic [46], so choosing an area much larger (such as geopolitical units, or areas based on vegetation) than the distribution of liolaemid species will cause unwanted situations: i.e: species that are allopatric having assigned the same area, or species that are present only in a small part of the area assigned to them, effectively overestimating the actual distributions.

This paper aims to evaluate the behavior of three common methods for ancestral area analysis: Fitch optimization, Weighted Ancestral Area Analysis and Dispersal-Vicariance Analysis, using as example the lizard family Liolaemidae, making a historical biogeography analysis that addresses the shortcomings of previous contributions, and using for the first time for this family, areas defined based on actual distributions rather than predefined or *ad hoc* areas.

## Materials and Methods

### Phylogeny

**Figure 1:**
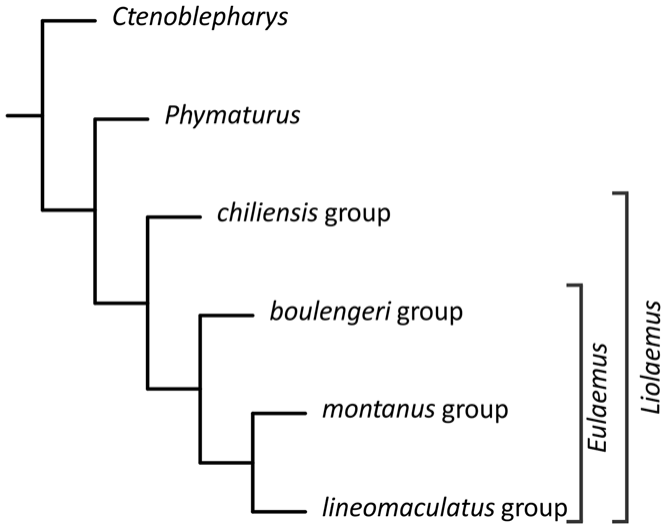
Cladogram depicting the phylogenetic relationships of the family Liolaemidae, constructed joining recent partial phylogenies for each of the clades included (called a Metatree). Each terminal, with the exception of *Ctenoblepharys* represent several species: *chiliensis* group, 86 species; *boulengeri* group, 40 species; *montanus* group, 9 species, *lineomaculatus* group, 12 species.

The ancestral area methods require a phylogeny of the taxa under study. However, to date there is no complete phylogeny of the three genera published. Recent molecular based phylogenies [29] found *Liolaemus* and *Phymaturus* as sister taxa, with *Ctenoblepharys* as sister to that clade. Following that the hypothesis from that study, a cladogram including the three genera was constructed (called a metatree [47]) (Fig. 1). For *Liolaemus*, recent phylogenies were used for the Chilean group [45], and Argentinian (Eulaemus) group [28], [29]. *Phymaturus* phylogeny was taken from Lobo & Quinteros [24]. *Ctenoblepharys* was then added as the most basal taxon. The total number of species included in the phylogeny is 170 (147 *Liolaemus, 22 Phymaturus* and *Ctenoblepharys*).

### Area selection

In order to improve over the previous contributions, an endemism analysis was made to delimit or describe area units to be used in the ancestral area analysis, based on actual Liolaemid distributions. For the endemism analysis, distributional data were collected for all the species included in the metatree, from museum collections and from theliterature. A data matrix was constructed and analyzed using the software NDM (Endems) [49], [50]. NDM searches for areas of endemism using an optimality criterion that includes a spatial component. NDM has been shown to outperform other common methods for identifying areas of endemism [49], [51], [52] such as Parsimony Analysis of Endemicity (PAE; [53]), that consists on scoring on a grid presences/absences of a set of species in a matrix, and then analyzing it under parsimony using the grids as terminals and the species as characters. Clades supported by two or more taxa are considered to represent areas of endemism. A grid size of 0.75°×0.75° was used; as there is no formal argument to select a ‘better’ grid size, different grid sizes were evaluated, and selected the size that produced more areas, defined by more taxa(with higher endemicity index). Grid origins were fixed at X = −80, Y = 5. Radius size used were: to fill: X = 40, Y = 40; to assume X = 80, y = 80. Searches for endemism areas were conducted using the following options: save sets with two or more endemic species, with score of 1.5 or higher; swap one cell at a time; discard superfluous sets; keep overlapping subsets only if 50% of species unique; use edge proportions. In order to improve the support of the areas found, twenty replicates of the analysis were made, each using a different seed number, and the resulting areas were saved in a file. Later, duplicate areas were deleted using the command ***‘d’***, and a consensus was calculated using a cut-off of 40% (percent of similarity in species), and including areas in the consensus only if it shares that percentage of similarity with all other areas in the consensus.

After the analysis, to each of the 170 species included in the phylogeny depicted in the metatree, an area unit was assigned following the results of the NDM analysis. The species that were not recovered as endemic to any area had to be assigned to one or more areas examining its distribution and comparing it to the areas found in the NDM analysis.

### Ancestral area analysis

For the ancestral area analysis three different methods were used: Fitch optimization [7], Dispersal-Vicariance Analysis (DIVA) [9] and Weighted Ancestral Area Analysis (WAAA henceforth) [8]. These methods use reversible parsimony to optimize the areas on a tree, finding an estimate of the ancestral distribution of a monophyletic clade, the ancestral area. Fitch optimization was proposed by Ronquist [7] as an alternative to the Camin-Sokal (irreversible) optimization proposed originally by Bremer [6]. DIVA uses a three-dimensional cost matrix that assigns different costs to events (extinctions, dispersals and vicariance) in order to minimize dispersal events. Using this approach, vicariance events have no cost, whereas dispersals and extinctions have a cost of one per area unit added to the distribution. The optimal reconstruction(s) are those requiring the minimal number of dispersal events. WAAA uses Fitch parsimony with a weighting scheme that weights favourably plesiomorphic areas, and areas more common as terminals. With this method, a probability index (PI) is calculated to give a measure of the likelihood of a particular area being part of the ancestral area.

For the DIVA analysis an additional adjustment was needed. Due to a limitation of the software [9] no more than 15 area units can be used in the analysis. In order to apply the method, the areas obtained by the NDM analysis were examined and areas which overlapped extensively (i.e. more than 50 percent) were joined forming one area to be used in the DIVA analysis.

Fitch optimization was made with the software TNT version 1.1 [54], constructing a matrix consisting on the tree and one character representing the distribution of the terminals. Weighted Ancestral Area Analysis was made with the help of an Excel spreadsheet, and Dispersal Vicariance Analysis was made with the software DIVA, version 1.2 [9]. The following options were used: settings: hold = 32767, weight = 1.000, age = 1.000.

Dispersal-evolution-cladogenesis model (DEC) was not applied in this study because the method requires molecular phylogenies to estimate likelihoods, and two of the phylogenies used (Chilean group of *Liolaemus* and *Phymaturus*) are strictly morphology-based, and are the most complete published to date, including more than half of the species included in this paper, making it impossible to evaluate this new and potentially useful approach to the estimation of ancestral ranges.

## Results

### Areas

**Figure 2:**
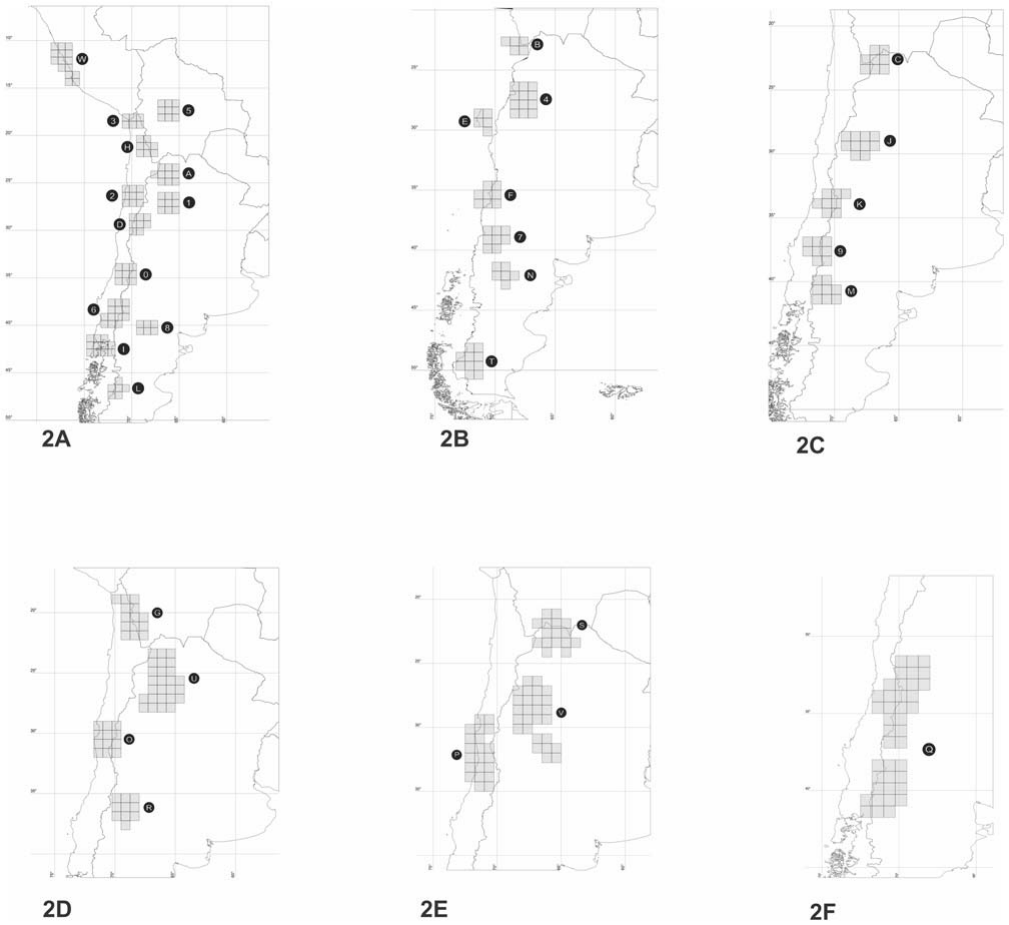
Areas found by NDM (Endems), a software to search and indentify areas of endemism. **2A:** 0: Central Chile (Maule, O'Higgins); 1: Cumbres Calchaquíes; 2: Atacama; 3: Arica; 5: Central Bolivia; 6: Araucanía and Bío Bío; 8: Central Río Negro; A: Prepuna of Salta and Jujuy; D: Cuyo; H: Northern Atacama (Chile).- I: Los Lagos; L: Central Patagonia (Santa Cruz); W: Coastal Central Peru. **2B:** 4: Prepuna of Catamarca; 7: Payunia; B: Atacama (Chile); E: Atacama and Coquimbo; F: Maule; N: Central Patagonia (Río Negro); T: Austral Patagonia. **2C:** 9: Payunia and Central Chile; C: Puna of Jujuy; J: Central Monte (La Rioja); K: Central Chile (Metropolitana, O'Higgins); M: Northern Patagonia. **2D:** G: Atacama and Puna of Bolivia; U: Sierras Subandinas and Cumbres Calchaquíes; O: Coquimbo; R: Central Monte (Mendoza). **2E:** S: Prepuna (Jujuy and Bolivia); V: Prepuna and Monte Boreal; P: Central Chile. **2F:** Q: Payunia and Monte Central.

The NDM analysis gave as a result 40 areas, and after the consensus procedure 32 remained (Table 1). These areas are shown in Figure 2. The areas used for the DIVA analysis are listed in Table 2. *Ctenoblepharys* is not represented in any of the original areas, a new area was defined corresponding to the distribution of this genus, in order to be able to include *Ctenoblepharys* in the analysis.

**Table 1:** Area codification.

#	Area	#	Area
**0**	Central Chile (Maule, O'Higgins)	**H**	Northern Atacama (Chile)
**1**	Cumbres Calchaquies	**I**	Los Lagos (Chile)
**2**	Atacama	**J**	Central Monte (La Rioja)
**3**	Arica	**K**	Central Chile (Metropolitana, O'Higgins)
**4**	Prepuna of Catamarca	**L**	Central Patagonia (Santa Cruz)
**5**	Central Bolivia	**M**	Northern Patagonia
**6**	Araucanía, Bío-Bío	**N**	Central Patagonia (Río Negro)
**7**	Payunia	**O**	Coquimbo
**8**	Central Río Negro	**P**	Central Chile (Coquimbo)
**9**	Payunia and Central Chile	**Q**	Payunia and Monte Central
**A**	Prepuna of Salta and Jujuy	**R**	Central Monte (Mendoza)
**B**	Atacama (Chile)	**S**	Prepuna (Jujuy and Bolivia)
**C**	Puna of Jujuy	**T**	Austral Patagonia
**D**	Cuyo	**U**	Sierras Subandinas and Cumbres Calchaquíes
**E**	Atacama and Coquimbo	**V**	Prepuna and Monte Boreal
**F**	Maule	**W**	Coastal Central Peru
**G**	Atacama and Puna of Bolivia		

The table show the letters used to represent different areas found by the endemism analysis.

**Table 2:** Areas for DIVA.

Original areas	DIVA areas	Original areas	DIVA areas
1-4-D-I	A	L	I
0-F-K-R	B	V	J
3-G-H	C	8	K
6-7-9-Q	D	I	L
10-B-C-S-U	E	C	M
2-E	F	5	N
M-N	G	Ctenoblepharys*	O*
O-P	H		

Original areas from the NDM analysis and resulting areas used in the DIVA analysis, obtained by joining together the original areas. For *Ctenoblepharys*, not represented in any of the original areas, a new area was defined as O.

### Ancestral area analysis

**Figure 3:**
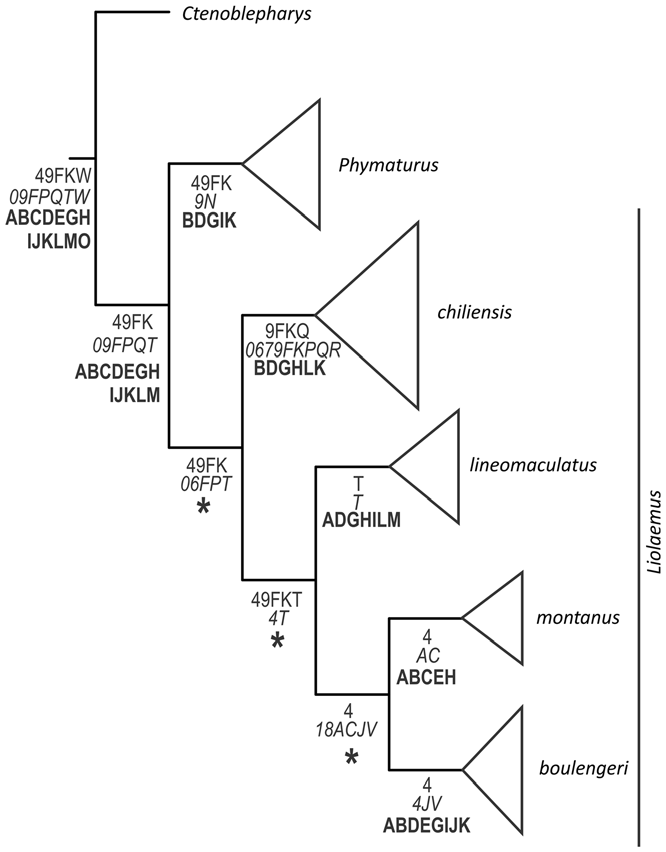
Cladogram of Liolaemidae with ancestral area assignations. The triangles indicate that each terminal represent several species. The numbers inside triangles show number of species. Normal: Fitch optimization, Italics: Weighted Ancestral Area Analysis. Bold: Dispersal Vicariance Analysis (DIVA). Nodes with an asterisk have multiple optimal reconstructions.

The ancestral area analyses were applied on the complete phylogenies at species level, optimizing the distribution of individual species, but for clarity, only the results at higher level clades are reported. All three methods (Table 3) recovered Central Chile and Payunia as part of the ancestral area for Liolaemidae. The area Coastal Peru also appeared as part of the ancestral area, yielding a disjunct ancestral area (Figure 3). Both Fitch optimization and WAAA recovered an ancestral area smaller than the distribution of Liolaemidae, which implies dispersalist explanations. DIVA on the other hand recovered an ancestral area almost equal as the actual distribution of Liolaemidae, preferring mostly vicariant explanations.

**Table 3:** Ancestral areas.

				Fitch	WAAA	DIVA
**Liolaemidae**				49FKW	09FPQTW	ABCDEGHIJKLMO
	*Ctenoblepharys*			W	W	W
	*Phymaturus*			49FK	9N	BDGIK
	*Liolaemus*			49FKQT	06FPT	*
		*Chiliensis*		9FKQ	0679FKPQR	BDGHLK
		*Eulaemus*		4T	4T	*
			*lineomaculatus*	T	T	ADGHILM
			*(montanus+boulengeri)*	4	18ACJV	*
			*montanus*	4	AC	ABCEH
			*boulengeri*	4	4JV	ABDEGIJK

Ancestral area assignations for Liolaemidae and included clades. Groups with an asterisk have multiple optimal reconstructions, listed in Table 4.

**Figure 4:**
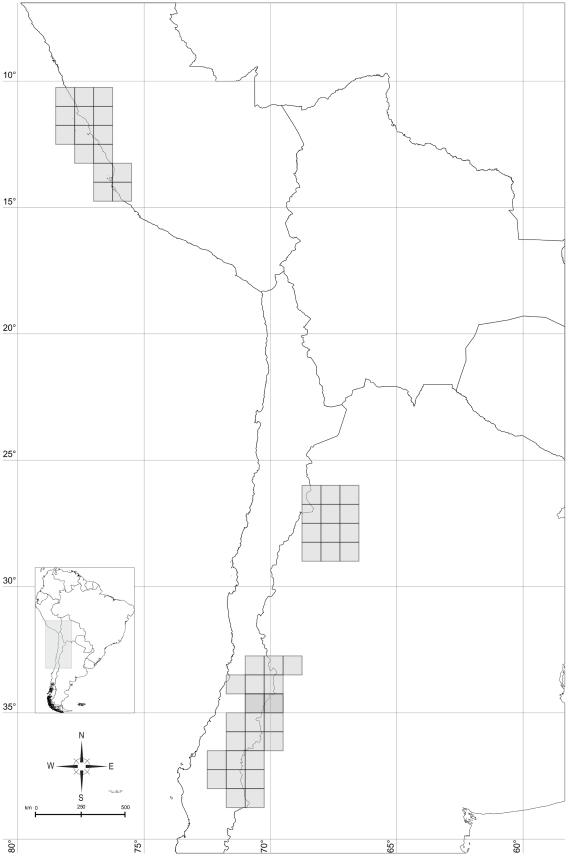
Ancestral area for Liolaemidae found by Fitch optimization. Numbers or letters refer to Fig. 2. Formed by: Prepuna of Catamarca (4), Payunia and Central Chile (9), Maule (F), Central Chile (Región Metropolitana and O'Higgins- K) and Coastal Perú (W).

**Figure 5:**
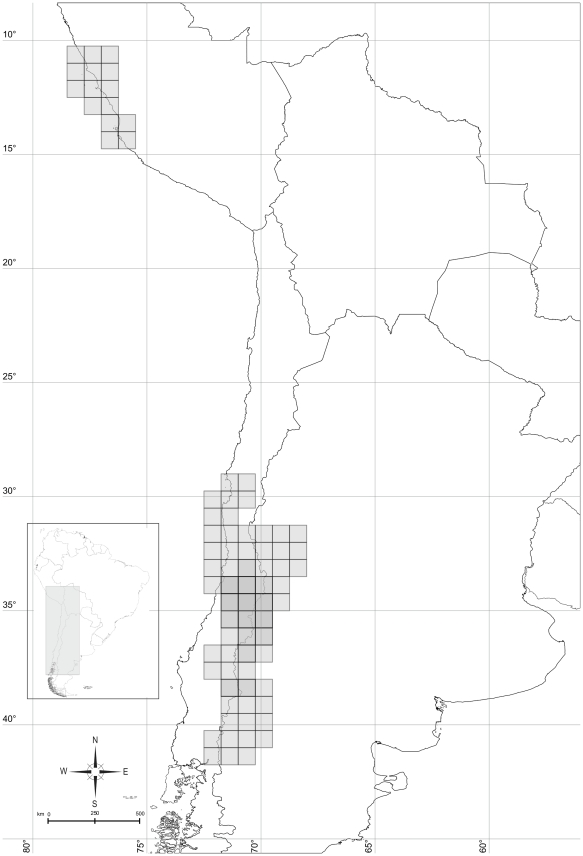
Ancestral area for Liolaemidae found by Weighted Ancestral Area Analysis (WAAA). Numbers or letters refer to Fig. 2. Formed by: Central Chile (0), Payunia and Central Chile (9), Maule (F), Coquimbo (P), Payunia (Q), Austral Patagonia (T), and Coastal Perú (W).

**Figure 6:**
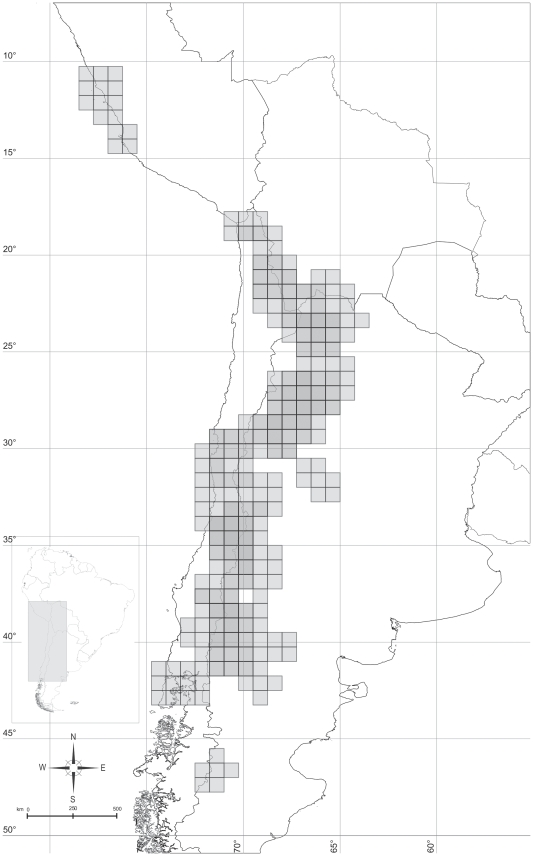
Ancestral area for Liolaemidae found by Dispersal Vicariance analysis (DIVA). Numbers or letters refer to Fig. 2. Includes all area units, except Atacama (B), Coquimbo (P) and Austral Patagonia (T).

Fitch optimization assigned to the family Liolaemidae as ancestral the following areas: Prepuna of Catamarca, Payunia and Central Chile, Maule, Central Chile (Región Metropolitana and O'Higgins) and Coastal Peru (corresponding to *Ctenoblepharys*) (Fig. 4). WAAA assigns to the family Liolaemidae (Fig. 5) an ancestral area formed by Central Chile, Maule, O'Higgins, Coquimbo, Payunia, Austral Patagonia and Coastal Peru. Dispersal Vicariance analysis assigned to the family Liolaemidae an ancestral area which encompasses almost all the actual distribution of the family, only Atacama, Coquimbo and part of Austral Patagonia are excluded (Fig. 6).

## Discussion

### About the methods

Fitch optimization may recover one or more areas as ancestral, as happens for Liolaemidae. However, the interpretation of these results is not direct. In the case of this study, Fitch optimization recovers four areas as ancestral, but these should not be interpreted as one ancestral area formed by four units, but as four equally probable ancestral areas. As such, Fitch optimization will produce an all-dispersal scenario, no matter how many area units are recovered as ancestral.

Regarding plesiomorphic areas being more likely part of the ancestral area, both Fitch optimization and WAAA give excessive importance to the basal position of a particular area in the cladogram. This is evident for the distribution area of *Ctenoblepharys* (Coastal Peru), which is the most basal area in the cladogram, and appears as part of the ancestral area for the family, rendering a disjunct ancestral area. The problem with disjuncts ancestral areas is that this disjunction has to be explained by deficient sampling or undetected extinctions, as one monophyletic clade cannot have originated in more than one area simultaneously, as would be the case of a disjunct ancestral area. Unsurprisingly, this is the same result as the one from the historical biogeography analysis of *Phymaturus* [16], even though in this study a complete phylogeny with more appropriate areas for *Liolaemus* was used. The area Coastal Peru will appear as part of the ancestral area in every reconstruction, even though it is the area of only one species, just because of its basal position, showing that this position of an area in the cladogram will outweigh any other criteria WAAA tries to solve the problem of giving excessive weight to plesiomorphic areas counting how many times a particular area appears in the cladogram. This way, an area that is not plesiomorphic can still be recovered as ancestral if is occupied by several taxa. However, in practice the most plesiomorphic area will have the highest probability index of all, making very difficult for other areas to reach a similar index.

Dispersal Vicariance Analysis uses reversible parsimony, but uses a cost scheme that favors vicariance, giving as a result ancestral area reconstructions that usually include most (if not all) of the areas, or giving several equally optimal reconstructions (Table 4; i.e. more than 100 reconstructions for *Eulaemus*). Ronquist [9] proposed two possible solutions: one is add more outgroups, making the basal or root node no longer root; and limit the maximum number of areas allowed to be part of the ancestral area. The first solution, at least for Liolaemidae, is difficult to implement. In the phylogenetic proposal of Frost and Etheridge [55] Liolaemidae is the most basal subfamily of Tropiduridae, but there is no consensus about its sister taxon, so adding outgroups for Liolaemidae is problematic. Even if outgroups could be added, if those outgroups were distributed on an area not occupied by Liolaemidae species, DIVA would add this new area to the ancestral reconstruction (as happens with the area of *Ctenoblepharys*). The second proposal allows restricting the maximum number of areas recovered as ancestral [16], [51]. However, there isn't a criterion to select a number of areas to restrict the ancestral area, and this procedure should be used carefully. Kodandaramaiah [19] suggested using different levels of constraint instead of no constraining, or constraining to two or three areas; and avoiding the use of DIVA when large scale extinctions are suspected, given that DIVA does not model extinctions well.

**Table 4:** Multiple reconstrucions for DIVA.

Node	Reconstructions
**(** ***boulengeri montanus*** **)**	EJ ACEJ BCEJ ABCEJ CDEJ ACDEJ BCDEJ ABCDEJ CEGJ ACEGJ BCEGJ ABCEGJ CDEGJ ACDEGJ BCDEGJ ABCDEGJ CEHJ ACEHJ BCEHJ ABCEHJ CDEHJ ACDEHJ BCDEHJ ABCDEHJ CEGHJ ACEGHJ BCEGHJ ABCEGHJ CDEGHJ ACDEGHJ BCDEGHJ ABCDEGHJ CEIJ ACEIJ BCEIJ ABCEIJ CDEIJ ACDEIJ BCDEIJ ABCDEIJ CEGIJ ACEGIJ BCEGIJ ABCEGIJ CDEGIJ ACDEGIJ BCDEGIJ ABCDEGIJ CEHIJ ACEHIJ BCEHIJ ABCEHIJ CDEHIJ ACDEHIJ BCDEHIJ ABCDEHIJ CEGHIJ ACEGHIJ BCEGHIJ ABCEGHIJ CDEGHIJ ACDEGHIJ BCDEGHIJ ABCDEGHIJ CEJK ACEJK BCEJK ABCEJK CDEJK ACDEJK BCDEJK ABCDEJK CEGJK ACEGJK BCEGJK ABCEGJK CDEGJK ACDEGJK BCDEGJK ABCDEGJK CEHJK ACEHJK BCEHJK ABCEHJK CDEHJK ACDEHJK BCDEHJK ABCDEHJK CEGHJK ACEGHJK BCEGHJK ABCEGHJK CDEGHJK ACDEGHJK BCDEGHJK ABCDEGHJK CEIJK ACEIJK BCEIJK ABCEIJK CDEIJK ACDEIJK BCDEIJK ABCDEIJK CEGIJK ACEGIJK BCEGIJK ABCEGIJK CDEGIJK ACDEGIJK BCDEGIJK ABCDEGIJK CEHIJK ACEHIJK BCEHIJK ABCEHIJK CDEHIJK ACDEHIJK BCDEHIJK ABCDEHIJK CEGHIJK ACEGHIJK BCEGHIJK BCEGHIJK CDEGHIJK ACDEGHIJK BCDEGHIJK ABCDEGHIJK
***Eulaemus***	ACEJM ABCEJM ACDEJM ABCDEJM ACEGJM ABCEGJM ACDEGJM ABCDEGJM ACEHJM ABCEHJM ACDEHJM ABCDEHJM ACEGHJM ABCEGHJM ACDEGHJM ABCDEGHJM ACEIJM ABCEIJM ACDEIJM ABCDEIJM ACEGIJM ABCEGIJM ACDEGIJM ABCDEGIJM ACEHIJM ABCEHIJM ACDEHIJM ABCDEHIJM ACEGHIJM ABCEGHIJM ACDEGHIJM ABCDEGHIJM ACEJKM ABCEJKM ACDEJKM ABCDEJKM ACEGJKM ABCEGJKM ACDEGJKM ABCDEGJKM ACEHJKM ABCEHJKM ACDEHJKM ABCDEHJKM ACEGHJKM ABCEGHJKM ACDEGHJKM ABCDEGHJKM ACEIJKM ABCEIJKM ACDEIJKM ABCDEIJKM ACEGIJKM ABCEGIJKM ACDEGIJKM ABCDEGIJKM ACEHIJKM ABCEHIJKM ACDEHIJKM ABCDEHIJKM ACEGHIJKM ABCEGHIJKM ACDEGHIJKM ABCDEGHIJKM ACEJLM ABCEJLM ACDEJLM ABCDEJLM ACEGJLM ABCEGJLM ACDEGJLM ABCDEGJLM ACEHJLM ABCEHJLM ACDEHJLM ABCDEHJLM ACEGHJLM ABCEGHJLM ACDEGHJLM ABCDEGHJLM ACEIJLM ABCEIJLM ACDEIJLM ABCDEIJLM ACEGIJLM ABCEGIJLM ACDEGIJLM ABCDEGIJLM ACEHIJLM ABCEHIJLM ACDEHIJLM ABCDEHIJLM ACEGHIJLM ABCEGHIJLM ACDEGHIJLM ABCDEGHIJLM ACEJKLM ABCEJKLM ACDEJKLM ABCDEJKLM ACEGJKLM ABCEGJKLM ACDEGJKLM ABCDEGJKLM ACEHJKLM ABCEHJKLM ACDEHJKLM ABCDEHJKLM ACEGHJKLM ABCEGHJKLM ACDEGHJKLM ABCDEGHJKLM ACEIJKLM ABCEIJKLM ACDEIJKLM ABCDEIJKLM ACEGIJKLM ABCEGIJKLM ACDEGIJKLM ABCDEGIJKLM ACEHIJKLM ABCEHIJKLM ACDEHIJKLM ABCDEHIJKLM ACEGHIJKLM ABCEGHIJKLM ACDEGHIJKLM ABCDEGHIJKLM
***Liolaemus***	ACEHJLM ABCEHJLM ACDEHJLM ABCDEHJLM ACEGHJLM ABCEGHJLM ACDEGHJLM ABCDEGHJLM ACEHIJLM ABCEHIJLM ACDEHIJLM ABCDEHIJLM ACEGHIJLM ABCEGHIJLM ACDEGHIJLM ABCDEGHIJLM ACEHJKLM ABCEHJKLM ACDEHJKLM ABCDEHJKLM ACEGHJKLM ABCEGHJKLM ACDEGHJKLM ABCDEGHJKLM ACEHIJKLM ABCEHIJKLM ACDEHIJKLM ABCDEHIJKLM ACEGHIJKLM ABCEGHIJKLM ACDEGHIJKLM ABCDEGHIJKLM

Clades with multiple optimal reconstructions found by DIVA. All reconstructions have the same cost, and are equally probable.

There are more things to consider about DIVA. The program is no longer maintained (it wasn't available to download from Ronquist's website at the time of writing this article), and also has some serious limitations: it cannot accept polytomies in the cladograms, the maximum number of taxa that can be included is 180, and no more than 15 area units can be used in the analysis. For Liolaemidae, the number of species currently is more than 240 [30], [31] and more are described each year. When updated phylogenies are published, it will not be possible analyze them with DIVA because of this limitation. The maximum number of areas allowed forces one to make *ad hoc* decisions, as joining areas to reach that number, which negates the advantage of make an endemism analysis to identify areas which more accurately reflect the distribution of the species included in the study.

For the analysis of the historical biogeography of a taxon, Fitch optimization should be avoided, unless a dispersalist explanation is preferred. Weighted ancestral area analysis and DIVA remain as alternatives, bearing in mind their limitations, particularly in the case of DIVA. Using fewer areas will facilitate the analysis and the interpretation of the results, but this may require compromises like joining areas together, or constraining the maximum number of areas recovered as ancestral.

### Liolaemidae

In a previous study of the historical biogeography of *Phymaturus*, Díaz Gómez [16] included two more terminals representing *Liolaemus* and *Ctenoblepharys*. In that study, the same methodologies used here were applied, but the area units used were those proposed by other authors and were based on arthropods [37]–[39]. Moreover, the area assigned to *Liolaemus* was not based on an explicit analysis, rather assigned based on a partial study of a subclade of *Liolaemus* [46] and paleontological data. In that study, Patagonia Central and Coastal Peru were proposed as ancestral areas for the family Liolaemidae. With the exception of the area in Peru, the ancestral areas found in this study do not include Patagonia Central.

The area Patagonia Central appeared in that study mainly because of its basal position on the cladogram. The ancestral area methods favor more plesiomorphic areas as part of the ancestral areas. Also, the area Patagonia Central as defined by Roig-Juñent *et al.* [39] also does not correspond with the areas found here for Liolaemidae, being awide area including most of the south of Argentina.

Fitch optimization founds a disjunct ancestral area. This could be interpreted in two ways: Only one of each of the areas is the ancestral area (as in character reconstruction), which implies an all-dispersal scenario, or accept a disjunct ancestral area. However, as a monophyletic group can only have one origin, one must assume that the disjunction is caused by extinction or lack of data. Also it is evident that the areas Coastal Peru and Prepuna of Catamarca are recovered mainly because of their basal position in the cladogram.

The results of this study can be resumed on two different and opposing hypotheses. One postulates a restricted ancestral area for the ancestor of Liolaemidae, located in Central Chile and Payunia, and the current distribution would be explained by dispersal to Patagonia and, following the Andes to the north including Puna and Prepuna. The paleontological evidence available is congruent with this hypothesis; the oldest and currently only fossil of a member of the Liolaemidae family is a *Liolaemus* from the Miocene of Patagonia, at the Gantman formation in Chubut, Argentina [56].

The other hypothesis (from DIVA analysis) postulates a widespread ancestor, distributed from Peru to the Patagonia, following the Andes and arid regions in South America (Fig. 5). The current distribution of the family would be explained by successive vicariant events that fragmented the distributions and dispersals in order to explain the species found in the east of South America. More paleontological data could be useful to support this explanation, but unfortunately there are no fossil records for *Ctenoblepharys* or *Phymaturus*. For Iguanidae the fossil record is scarce [53], the earliest fossil that can be referred to Iguanidae is *Pristiguana brasiliensis*, from the Upper Cretaceous Baurú formation of Brazil [58]. Interestingly, this fossil shows characters similar to the tropidurines, a clade closely related to Liolaemidae [25]. There are other records for Iguanian lizards for the Cenozoic of Bolivia [59] and Patagonia [60]. If these fossils could be related to ancestors of Liolaemidae, they would support a widespread origin for the family.

Some aspects of the distribution of Liolaemidae could be explained by a widespread ancestor. For example, most *Liolaemus* species are distributed in the arid regions of southern South America, and in the Andes cordillera, precordillera and Patagonia. However, a small group of species (of the *weigmannii* group) are distributed forming a series of ‘islands’ from the coasts of Buenos Aires, Uruguay and Brazil, up to Rio de Janeiro, associated with sand dunes. These species are disjunct from the rest of *Liolaemus* species that are not present in the Chaco or in humid forests. The desertification process from the Miocene to Pliocene [57] may have allowed these sand systems to expand, followed by the expansion of the distribution of the ancestors of those species. After that, the arid/humid cycles following the glacial and interglacial periods of the Pliocene and Pleistocene produced expansions and retractions of humid and xeric habitats, acting as vicariant events and causing the fragmentation and speciation of the extant taxa [61]–[64].

### About *Phymaturus*

Cei [21] postulated an Andean-Patagonian origin for *Phymaturus*, based on a refuge theory, where the patagonic tablelands would have been refuges and neo-dispersal centres for the iguanian fauna of Patagonia, particularly for *Liolaemus* and *Phymaturus*. Later, Pereyra [34], based on a phenetic analysis, followed Cei's hypothesis proposing Patagonia as the centre of origin for *Phymaturus*, postulating a differentiation between northern and southern populations, and a posterior invasion to the Patagonia by the northern species. Neither of these hypotheses are supported by the results of this study, that postulate as ancestral area for *Phymaturus* Payunia, Central Chile and northern Patagonia. In the case of the scenario proposed by Pereyra, only Fitch optimization includes part of the northern distribution of *Phymaturus* (Prepuna of Catamarca), but there are no dispersals of the northern species to southern areas.

Díaz Gómez [51] published an ancestral area analysis for *Phymaturus*, applying the same methodology used here, but using areas defined by arthropods [36]. In that study the ancestral area for *Phymaturus* was Central Patagonia (plus Andean Cordillera and Valle Central in Chile for DIVA analysis). The ancestral area found here by Fitch optimization is not congruent with those results. The WAAA results of this study are congruent with Díaz Gómez (2007), including Central Chile and Central Patagonia. However, the area identified as Patagonia in the previous study is much bigger than the area defined here as Central Patagonia, making the results not directly comparable. The DIVA results of this study are congruent with the area proposed by Díaz Gómez [48], mainly because DIVA found an ancestral area that encompass almost all the current distribution of *Phymaturus*, including completely the areas Payunia, Central Chile, Central Patagonia and Araucanía.

### About *Liolaemus*

Laurent [65]–[67] divided the genus *Liolaemus* in two groups, the Chileno group (*Liolaemus sensu stricto* or *chiliensis*) and the Argentino group (*Eulaemus*), pointing at the Andean uplift as the cause of this division. The results from this study support this hypothesis, because the ancestral area of the *chiliensis* group includes areas mainly west of the cordillera (Central Chile, Coquimbo, southern Chile), and the Argentino group includes areas east of the cordillera (Prepuna of Catamarca, Monte Central en La Rioja, and south of Patagonia).

Díaz Gómez & Lobo [45] made an ancestral area analysis for the *chiliensis* group, using the same methods used in the present study, and the areas proposed by Roig-Juñent [43]. In that analysis, the area Andes was proposed as ancestral for the *chiliensis* group, with Fitch optimization and WAAA adding Monte. The results from this study are congruent with the previous proposals, although the ancestral area found here is bigger, including the ancestral area found by Díaz Gómez and Lobo [45], plus some areas not found as ancestral in that study, like areas in Chile. The area Monte is not recovered as ancestral in the analysis here presented, because the area was not found by the endemism analysis, and could not be included in the analysis.

Although there are some previous contributions for the historical biogeography of *Liolaemus* which included the *Eulaemus* or Argentino group [41], [44], none of those studies was focused on *Eulaemus* or included few species of this group. This is the first study of the historical biogeography of *Eulaemus* including a complete sample of species and recent phylogenies. Both Fitch optimization and WAAA found Prepuna of Catamarca and Austral Patagonia as ancestral area for the group. This disjunct ancestral area could be explained by the basal position within *Eulaemus* of the *lineomaculatus* group of species, mainly distributed in austral Patagonia. DIVA found as ancestral area almost all the extant distribution of the group.

This study estimates the ancestral area for Liolaemidae and its main clades, using three different methodologies and showing some limitations of the methods available to the study of ancestral areas. Cladistic biogeography studies will only be as good as the phylogenies they use. When more complete phylogenies are published, including new taxa (for example species distributed in the Atlantic coast of Brazil) the results of this study should be revised, and perhaps updated. So far, this is the first study with a complete sample of species and an important step for understanding the historical biogeography of this clade of lizards.
